# An outbreak of Legionnaires’ disease linked to a municipal and industrial wastewater treatment plant, The Netherlands, September–October 2022 

**DOI:** 10.2807/1560-7917.ES.2024.29.20.2300506

**Published:** 2024-05-16

**Authors:** Roan Pijnacker, Petra Brandsema, Sjoerd Euser, Ali Vahidnia, Arnold Kuiter, Jesse Limaheluw, Christine Schout, Gaaled Haj Mohammad, Stijn Raven

**Affiliations:** 1 Centre for Infectious Disease Control, National Institute for Public Health and the Environment (RIVM), Bilthoven, the Netherlands; 2 Department of Infectious Diseases, Public Health Service region Utrecht (GGD), Zeist, the Netherlands; 3 Regional Public Health Laboratory Kennemerland, Haarlem, the Netherlands; 4 Environmental Services Regarding Authorization and Enforcement (RUD Utrecht), Utrecht, the Netherlands

## Abstract

Wastewater treatment plants (WWTPs) are increasingly identified as Legionnaires’ disease (LD) sources. An outbreak investigation was initiated following five LD cases reported in September 2022 in Houten, the Netherlands. Case identification was based on the European LD case definition, with symptom onset from 1 September 2022, residence in or within 5 km of Houten, or visit to Houten within the incubation period, without other likely sources. We sampled potential sources and genotyped environmental and clinical isolates. We identified 15 LD cases with onset between 13 September and 23 October 2022. A spatial source identification and wind direction model suggested an industrial (iWWTP) and a municipal WWTP (mWWTP) as potential sources, with the first discharging water into the latter. Both tested positive for *Legionella pneumophila* serogroups 1 and 6 with multiple sequence types (ST). We detected *L. pneumophila* sg1 ST42 in the mWWTP, matching with one of three available clinical isolates. Following control measures at the WWTPs, no further cases were observed. This outbreak underlines that municipal and industrial WWTPs can play an important role in community LD cases and outbreaks, especially those with favourable conditions for *Legionella* growth and dissemination, or even non-favourable conditions for growth but with the influx of contaminated water.

**Key public health message**

**What did you want to address in this study and why?**

*Legionella* are bacteria that can cause a serious lung infection known as Legionnaires’ disease. People can get infected when they breathe in tiny water droplets contaminated with the bacteria. In September and October 2022, 15 cases of LD were reported in and around the town of Houten, the Netherlands. An investigation was started to find and control the source of infection.

**What have we learnt from this study?**

We identified two wastewater treatment plants (WWTPs) as the source. The first had warm and nutrient-rich water, favourable for *Legionella* growth, and discharged the water into the second WWTP. Although this second WWTP did not have optimal conditions for *Legionella* growth, it probably released tiny water droplets contaminated with the bacteria into the air. After control measures were taken in the plants, no further cases were reported.

**What are the implications of your findings for public health?**

This outbreak highlights the potential public health risks of WWTPs. This is especially the case for WWTPs with favourable conditions for *Legionella* growth. However, even when they do not have favourable conditions, they may still pose a health risk when they have influx of *Legionella*-contaminated water.

## Background

Legionnaires’ disease (LD) is a bacterial infection mostly caused by *Legionella pneumophila* species. The disease is characterised by pneumonia, often requires hospitalisation and in the Netherlands has a case fatality of ca 5% [1,2]. *Legionella pneumophila* is divided into 16 serogroups, among which *L. pneumophila* serogroup 1 (sg1) is responsible for ca 90% of diagnosed cases in Europe [3]. The incubation period is usually 2–10 days and rarely exceeds 14 days. *Legionella* bacteria are ubiquitous in the natural environment and can sometimes grow rapidly in man-made water systems. They can cause infection when inhaled after aerosolisation.

Although the majority of LD cases are sporadic, outbreaks are commonly reported, most often related to wet cooling towers, building water systems and spa pools [4,5]. Wastewater treatment plants (WWTPs) have increasingly been identified as a source in outbreaks of LD, but their role in sporadic LD is probably still underestimated [6]. Wastewater treatment plants with biological treatment systems may have an ideal temperature for *Legionella* growth, and the availability of oxygen and organic nitrogen can further enhance the proliferation of *Legionella* [6]. The aerobic treatment process generates aerosols that may contain *Legionella* that are spread to the environment. It is generally believed that industrial WWTPs (iWWTP) are more likely sources of infections than the traditional municipal WWTPs (mWWTP) due to their higher operating temperatures, often 30–38 °C, combined with nutrient-rich wastewater. Nonetheless, *Legionella* is prevalent in both industrial and municipal WWTPs as documented in several studies [7-9]. In the Netherlands, an industrial biological WWTP was identified as a common source for two clusters of LD cases in 2016 and 2017, and another iWWTP as a source for cases from 2013 to 2018 [10,11]. Since then, there have been multiple smaller clusters that were linked to WWTPs [12,13].

## Outbreak detection

In the period 19–28 September 2022, five cases of LD were reported to the Municipal Health Service (MHS) region of Utrecht; all were residents of the town of Houten, which has 46,970 inhabitants. Because no cases of LD had been reported in the previous 5 years among Houten residents and none of the five cases reported a likely source of exposure to aerosols, an outbreak investigation was initiated on 30 September, with the aim to identify the source of the outbreak and implement control measures. The team included epidemiologists, medical doctors in communicable disease control, an infection control specialist, environmental and public health policy advisors and microbiologists from the MHS region of Utrecht, the National Institute for Public Health and the Environment (RIVM), environmental authorities and the national reference laboratory for *Legionella* (NRLL). 

We describe here the epidemiological and environmental investigations that followed, including patient interviews, typing of clinical isolates, environmental sampling and modelling that together helped identify the most likely source of infection.

## Methods

### Surveillance

Legionnaires’ disease is a notifiable disease in the Netherlands. For a detailed description of the surveillance system, we refer to a previous publication [14]. In short, all diagnosed LD cases are reported to the MHS, who report the case to the national level (RIVM) via an online notification system. Communicable disease nurses of the MHS interview all cases using a standardised questionnaire on possible sources of aerosol exposure in the 2 weeks before disease onset and add this exposure information to the notification system. The list of potential sources includes e.g. travel, stay in a hospital or healthcare facility, visits to risk locations such as wellness facilities and pools, occupational exposure and activities such as gardening. In addition, exposure to wet cooling towers and wastewater treatment plants are considered for local clusters or outbreaks. Medical microbiological laboratories send clinical isolates to the NRLL, and a selection of potential environmental sources is sampled [14]. Sampling and typing of clinical and environmental isolates is done by the NRLL.

### Case definition and finding

For this outbreak, a confirmed LD case was defined as a patient with pneumonia and microbiological confirmation according to the European probable or confirmed LD case definition [15] and with symptom onset on or after 1 September 2022, living in the town of Houten or within 5 km of Houten, or who had visited Houten within the incubation period, without other likely sources. A patient with only a single high titre for *L*. *pneumophila* sg1–6 was defined as a suspected case, and paired samples would be required to classify the case as probable. To increase case finding, the MHS informed general practitioners in Houten about the LD increase through a digital letter on 4 October, encouraging them to perform diagnostics in patients with LD-like symptoms.

### Epidemiological investigations

For this outbreak, the MHS re-interviewed the cases, collecting information on recent movements in- and outside Houten (e.g. cycling, hiking, shopping). Furthermore, 6-digit postal codes of identified cases were entered in the LD-GIS-tool from the European Centre for Disease Prevention and Control (ECDC) (https://legionnaires.ecdc.europa.eu/gistool) to calculate a disease risk map based on the case density and population density for 2 km, 5 km and 10 km distance [16]. This information was used to generate hypotheses on the location of the infection source.

### Environmental investigations

To identify the source of this outbreak, possible locations for inspection and environmental sampling were identified in a radius of 5 km in or around Houten. To find registered wet cooling towers and WWTPs, we consulted the local environmental authority, examined the Atlas Living Environment maps which contain registered wet cooling towers [17], and visually inspected satellite images from Google Maps. We also reviewed the source finding interviews to identify possible common exposures. The *Legionella* Source Identification Unit from the NRLL took environmental samples from each of the possible source locations. Moreover, we consulted the environmental authority on locations with recent changes in operating procedures and technical failures that could have facilitated *Legionella* proliferation.

### Microbiological investigations

All collected environmental and clinical isolates were genotyped using sequence-based typing and compared with the European Working Group for *Legionella* Infections sequence-based typing database [18]. To increase typing resolution, molecular serogroups, multilocus sequence typing (MLST) sequence types (STs) and 1,521 locus cgMLST complex types (CTs) were calculated in Ridom Seqsphere+ software v7.7.5 by automated allele submission to the *Legionella pneumophila* cgMLST server (https://www.cgmlst.org/ncs/schema/schema/1025099) [19]. The allelic profiles were used to calculate distance matrices using a Hamming distance, ignoring pairwise missing loci.

We extracted DNA from cultured isolates using a robotic system MagCore extractor system H16 with a MagCore Viral extraction kit (RBC Bioscience). Sequencing libraries were prepared using the NextEra XT library prep kit (Illumina) and then run on miniSEQ Illumina platform using a 150 bp paired-end sequencing Mid output Kit v2 (Illumina). The acceptance criteria were set as percentage good targets > 90% and average coverage (assembled) > 30. Ridom SeqSphere+ was used to convert the cgMLST scheme developed by Moran-Gilad et al. [20]. The allelic profile output was used to create minimum spanning trees (NJ tree) that were based on 1,535 core genes including the seven household genes for sequence-based typing of MLST and 1,521 cgMLST. The cgMLST results of randomly selected human (n = 8) and environmental isolates (n = 1) sampled in 2020 and 2021 were added to the minimum spanning tree for context.

### Statistical analyses

Transmission of *Legionella* has been described over a long distance up to 12 km, and in a previous WWTP-associated outbreak in the Netherlands, transmission occurred over a distance of at least 3 km, with an increased attack rate up to 6 km distance [11,21,22]. Furthermore, the outcome of the Legionnaires GIS toolkit showed similar results for the 10 km and 5 km distance models. Therefore, we used both 10 km and 5 km distances in our models. We assumed that exposure most probably took place at the residential address, where most time is spent, as previously described [23].

Firstly, we used a spatial source identification model that has been described previously [10,24]. In short, it divides the area in a spatial grid and keeps the grid cells within a specified radius of a case. Each centre point of a cell is a potential source, with a number of cases assigned to them. The model fits an exponential decay function to the incidence–distance data for each cell. If this fit is significant at a 95% confidence level, the grid cell is retained as a potential source and a normalised measure of risk (nMR) is ca­­lculated. The measure of risk is the integral of a probability of illness function, which considers a baseline infectivity and distance (decay). This means that it takes into account the number of cases but also the population density, as well as the distance. This measure is normalised for comparison. The nMR has a value between 0 and 1, where a value closer to 1 indicates a more likely source. We assigned LD cases to a square location based on the postal code of their residential address. We first ran the model for a 1 × 1 km grid and a 10 km search radius, and then repeated the procedure for a 500 × 500 m grid with a 5 km search radius.

Secondly, we assessed whether upwind direction was correlated with the direction of each potential source location. We calculated the bearing (i.e. measure of direction, expressed here as degree of angle) between each potential source location and the cases’ residential address. For example, a bearing of 45 degrees means the potential source is north-east of the cases’ residence. We compared this bearing with the wind direction during the patients’ incubation period (2–10 days before disease onset) and calculated the difference in degrees between them. For example, when a potential source location was at 30 degrees from the cases’ residential address and the wind direction was 20 degrees, the difference would be 10 degrees. By chance a random distribution of 90 degrees difference would be expected. A two-tailed Student’s t-test was performed to determine whether this difference was significantly lower than expected by chance. The analyses were repeated weighting for wind velocity (Beaufort scale, with higher weight with increasing scale) and day of the incubation period (weight for day 2 to day 10: 0.048, 0.077, 0.125, 0.173, 0.202, 0.125, 0.125, 0.087, 0.038) [25]. We also performed the same analyses with the locations most reported as visited by cases instead of their residential address. Data on daily average wind direction in degrees and velocity (m/s) were obtained from a weather station located at ca 8.5 km of Houten via the Royal the Netherlands Meteorological Institute (KNMI, www.knmi.nl). Data on the distribution of the incubation period were obtained from a large LD outbreak in Melbourne, Australia [26]. 

All analyses were performed in RStudio v2022.07.2. The R package TrackReconstuction version 1.3 and the weighted Student’s t-test was performed using R package weights version 1.0.4. We used the CalcBearing function in the TrackReconstruction package to obtain the radian between a given initial latitude and longitude (potential source location) and ending latitude and longitude (residential address of a case) in decimal degrees. Radians were converted to degrees by multiplying by 180/π. Statistical significance was set to a p value of < 0.05.

## Results

### Descriptive epidemiology

**Figure 1:**
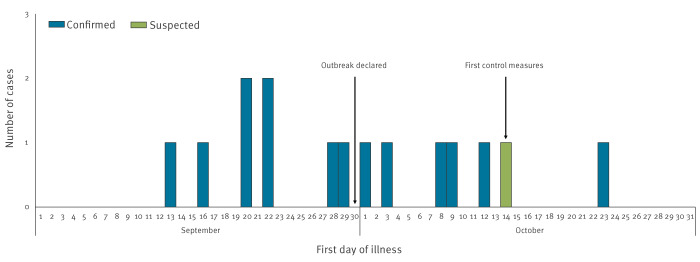
Number of confirmed and suspected Legionnaires’ disease outbreak cases by day of illness onset, Houten, the Netherlands, September–October 2022 (n = 15)

In total, 15 cases were identified, of whom 14 were confirmed and one was suspected. Disease onset ranged from 13 September to 23 October 2022 (Figure 1). Nine cases were female and six were male, with a median age of 65 years (range: 41–79 years). Nine patients had underlying health conditions, one case currently smoked, and for three information on risk factors was not given. All cases except one were admitted to hospital, one of them to the intensive care unit. No deaths were reported. Two cases reported travel abroad: for one of them, travel was considered an unlikely source because the case had travelled more than 10 days before disease onset and stayed in Houten during the entire 10-day incubation period. The other case had travelled abroad during the 4 days before onset of symptoms, and infection abroad could not be excluded. The only common exposure identified from case interviews was buying groceries at the same shopping mall (nine of 15 cases); six of the nine cases frequented the same supermarket, which had a mist system. Only one case reported a well-known LD risk exposure, which was a swimming pool. The only two patients not living in Houten reported to have visited Houten during their incubation period. Both cases lived within a proximity of 5 km of Houten and had visited the shopping mall that was also most often reported by other cases. Thirteen cases tested positive in the urine antigen test, of whom three were culture-positive in sputum. Two cases tested negative in the urine antigen test, but one of them tested positive in PCR on bronchoalveolar lavage and the other was single IgM antibody-positive for *L. pneumophilia* serogroup 1–6.

Possible source locations that were identified included a fountain, a demonstration of the fire brigade on 10 September 2022, the mist system in a supermarket where many cases purchased their groceries, a waste-processing company using water dispersion to minimise dust formation, an iWWTP and a mWWTP. No wet cooling towers were identified in or around Houten. The outcome of the LD-Gis tool indicated that the source was most likely to be located in the south or south-western region of Houten, corresponding to the locations of the waste-processing company, the iWWTP and the mWWTP.

### Environmental and microbiological investigations

**Figure 2:**
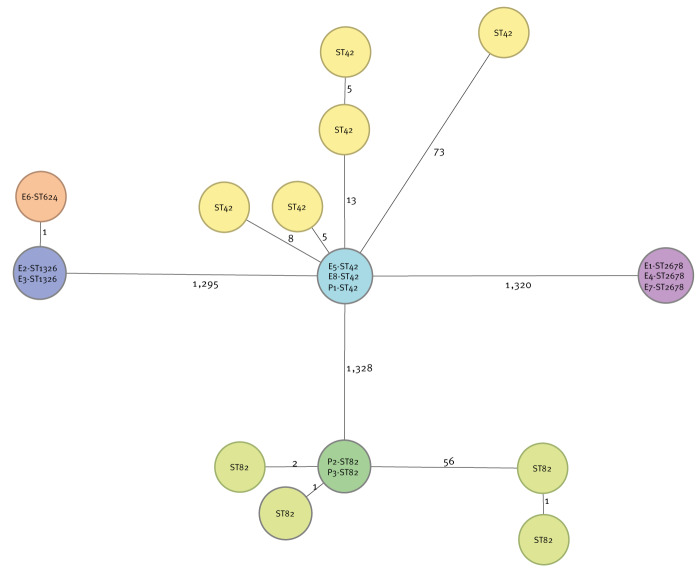
Minimum spanning tree based on 1,521 core genome sequencing typing targets of clinical (n = 3) and environmental (n = 8) isolates related to the Legionnaires’ disease outbreak in Houten, the Netherlands, September–October 2022, as well as randomly selected non-outbreak isolates for context of ST42 (n = 5) and ST82 (n = 4)

Clinical isolates were available for three cases and typed as *L. pneumophila* serogroup 1 ST82 in two cases and *L. pneumophila* serogroup 1 ST42 in one case. The two case isolates with ST82 were identical to each other based on cgMLST results, and closely related to two of four non-outbreak ST82 patient isolates that were included in the cgMLST analysis for context, with one and two alleles difference, respectively (Figure 2). No *Legionella* was detected in the environmental samples taken from 4 to 7 October 2022 from the fountain, fire brigade, waste-processing company and the supermarket mist system, making them less likely sources of infection.

iWWTP: industrial wastewater treatment plant; mWWTP: municipal wastewater treatment plant; ST: sequence type.

Outbreak isolates were from the iWWTP pulsewater (E1 and E2) and aeration tank (E3 and E4), the mWWTP aeration tank (E5 and E6), settling basin (E7) and effluent (E8), as well as three patients (P1–P3). The randomly selected isolates from human (n = 8) and environmental (n = 1) origin were sampled in 2020 and 2021.

Samples taken at the iWWTP and mWWTP on 4 and 7 October 2022, respectively, tested positive for *L. pneumophila*, with high concentrations between 2,000 and 20,000,000 colony-forming units (cfu)/L (Table 1). Samples from both locations were typed as *L. pneumophila* sg1, ST2678, which did not match with the typing of the clinical isolates. However, these isolates were identical to each other based on cgMLST. Both WWTPs also tested positive for *L. pneumophila* sg6 and in two samples from the mWWTP, *L. pneumophila* serogroup 1, ST42 was detected. The latter matched the typing results of one LD case, marking the mWWTP as a likely source of infection. This was corroborated by the cgMLST results which showed that the ST42 strains from the patient and the mWWTP were identical. Furthermore, five non-outbreak ST42 patient isolates had an allelic distance of five to 73 alleles with the outbreak ST42 isolates.

**Table 1:** Microbiological findings in environmental samples taken at possible source locations, Houten, the Netherlands, September–October 2022 (n = 13)

Location	Type of sample	Label^a^	Sampling date	Concentration (cfu/L)^b^	Serogroup	Sequence type
Fountain	Basin surface swab and water	NA	4 Oct	Not detected		
Fire brigade	Canal used for water intake	NA	4 Oct	Not detected		
Supermarket	Swab mist machine	NA	7 Oct	Not detected		
Waste-processing company	Reservoir spray water	NA	4 Oct	Not detected		
Industrial wastewater treatment plant	Pulsewater	E1	4 Oct	9,800,000	SG1	2,678
		E2			SG6	1,326
		NA	4 Oct	Not detected		
	Aeration tank	E3	4 Oct	20,000,000	SG6	1,326
		E4			SG1	2,678
	Effluent	NA	4 Oct	Not detected		
Municipal wastewater treatment plant	Aeration tank	E5	7 Oct	10,000,000	SG1	42
		E6			SG6	624
		NA	7 Oct	1,000,000	SG1	Unknown
	Settling basin	E7	7 Oct	2,000	SG1	2,678
	Effluent	E8	7 Oct	20,000	SG1	42
	Influent	NA	7 Oct	Not detected		

cfu: colony-forming units; NA: not applicable.

^a^ Labels correspond to the isolate in Figure 2.

^b^ Not detected means that results were below the detection limit, which varies per sample type.

### Statistical models

**Figure 3:**
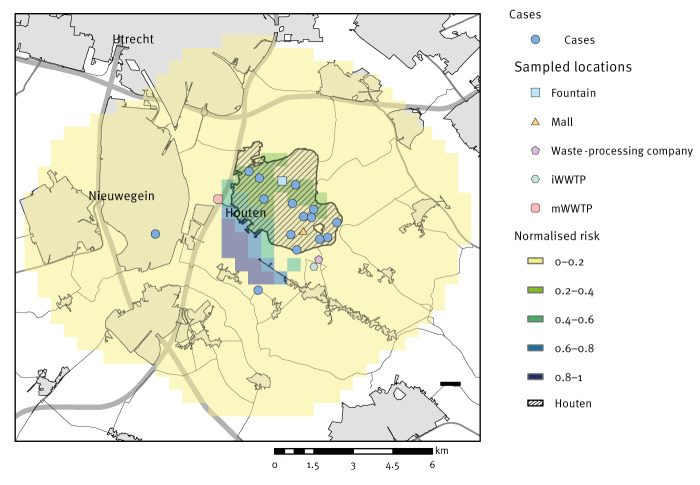
Map of normalised measure of risk for Legionnaires’ disease based on the residential address of confirmed and suspected Legionnaires’ disease cases, Houten, the Netherlands, September 2022–October 2022 (n = 15)

We used a spatial source identification model to determine whether the LD incidence decreased with an increasing distance from the centre of each possible source location (Figure 3). We included all 15 cases in the analyses because they lived within a 5 km radius of Houten. The fire brigade demonstration that was held on 10 September was excluded as possible source location because it could not explain cases that occurred after the maximum incubation period was exceeded. Based on these results, the most likely source was located south-west of Houten, where there were three putative source locations, namely the iWWTP, the mWWTP and the waste-processing company.

iWWTP: industrial wastewater treatment plant; mWWTP: municipal wastewater treatment plant.

The measure of risk is calculated using a spatial source identification model with a 5 km search radius. The residential location of the cases are plotted, as well as the possible source locations, including a fountain, mall, waste-processing company, iWWPT and mWWPT.

**Figure 4:**
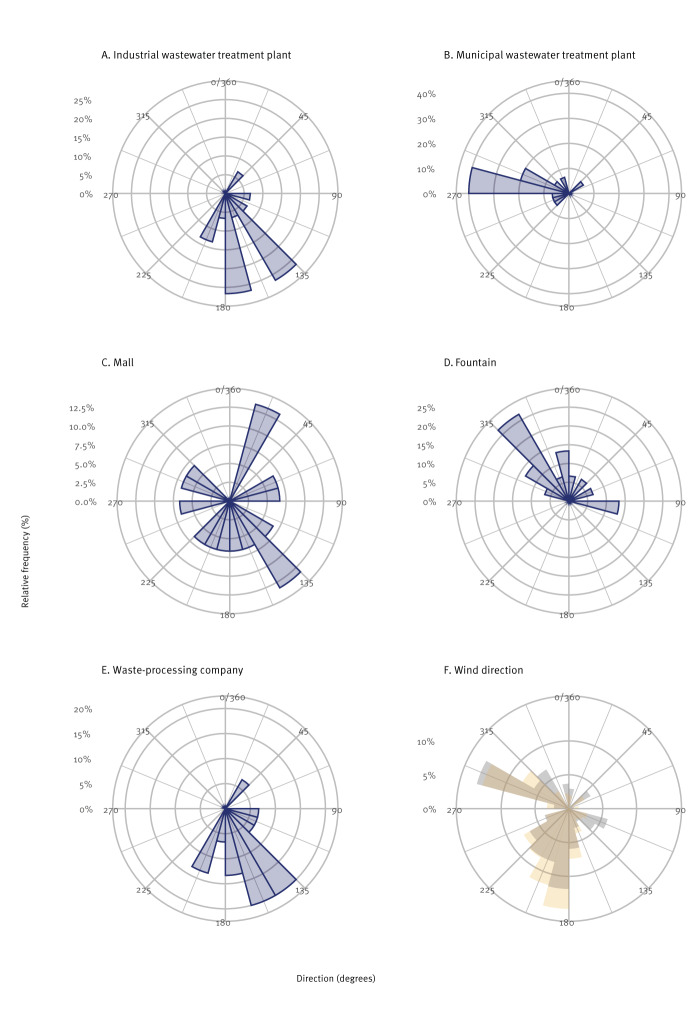
Rose plots of the direction of each possible source locations from the residential address of Legionnaires’ disease cases, indicated by the centre of the rose plot, Houten, the Netherlands, September–October 2022 (n = 15)

We used a wind direction model to assess whether any of the possible source locations were in line with the wind direction during the incubation period of the patients (Table 2 and Figure 4). The predominant wind direction during the outbreak was south/south-west/west. The mWWTP was most in line with the upwind direction (71.0° difference, p < 0.001), followed by the mall (80.1°, p = 0.018). When weighing for wind speed and incubation period, this difference was even smaller for the mWWTP (65.9°, p < 0.001) and remained the same for the mall (79.8°, p = 0.012). The maximum distance from any of the residential addresses of patients to the mWWTP was 4,8 km and 6,1 km to the iWWTP. However, the maximum distance from any of the residential addresses to either of the two WWTP was 3.0 km. The maximum distance to the mall was 5.6 km. We performed the same analyses using the mall as the location of exposure instead of the cases’ residential address, as this was the only commonly reported visited location. However, the results were all non-significant.

**Table 2:** Difference between the wind direction during the patients’ incubation period and the direction of each possible source location for confirmed and suspected Legionnaires’ disease cases, Houten, the Netherlands, September–October 2022 (n = 15)

	Unweighted			Weighted^a^		
	Mean difference	95% CI	p value^b^	Mean difference	95% CI	p value^b^
Fountain	96.9°	87.9–105.9	0.134	100.1°	91.9–108.4	0.017
Mall	80.1°	72.0–88.3	0.018	79.8°	72.0–87.6	0.012
Waste-processing company	85.8°	76.9–94.7	0.353	84.4°	75.6–93.3	0.222
iWWTP	84.0°	75.0–93.0	0.192	81.3°	72.4–90.3	0.060
mWWTP	71.0°	62.6–79.5	< 0.001	65.9°	57.9–74.0	< 0.001

CI: confidence interval.

^a^ Weighed for day of incubation period and wind speed.

^b^ Two-tailed Student’s t-test, with a null-hypothesis of 90°

iWWTP: industrial wastewater treatment plant.

Panels A–E: direction from the patients’ residential address. For example, rose plot A of the iWWTP indicates the direction to the iWWTP from the residential address of all patients. The y axis indicates the relative frequency of a certain direction being measured. In this case, for example, for 26.7% of patients, the iWWTP was located between 135° and 150° from their residential address.

Panel F: upwind direction profile during the incubation period of 2–10 days before disease onset of all patients (grey) as well as weighted for wind speed and the day of the incubation period (yellow). For example, the (unweighted) upwind direction was between 180° and 195° during 11.9% of days of the incubation period of all patients.

## Outbreak control measures

Based on the elevated concentration of *Legionella* at the iWWTP, as well as the first results of the spatial source identification model that pointed towards the iWWTP as the most likely source of infection, control measures were taken. Moreover, the environmental agency reported that the wastewater treatment process of the iWWTP had added an anaerobic treatment step to the wastewater treatment process 1 year before the outbreak, which increases the temperature of the wastewater, from ambient temperature to 30–38 °C. The aeration tank at the iWWTP was shut down on 14 October 2022 in order to prevent aerosol production that would facilitate the spread of *Legionella*. From 7 November 2022 onwards, effluent was treated with ultraviolet (UV) light to kill microorganisms and reduce the discharge of *Legionella* from the iWWTP to the mWWTP. The aeration tank of the mWWTP could not be shut down because of the oxygen requirement of the microorganisms that break down the wastewater pollutants. Because the mWWTP discharges into the Amsterdam–Rhine canal, this could potentially lead to contamination with wastewater pollutants. However, visitors were no longer allowed to enter the mWWTP and employees were required to wear a facemask. To prevent *Legionella* transmission, the aeration tank of the mWWTP was partially covered at the end of November 2023, but full coverage was not possible for practical and financial reasons. No further cases were observed after the aeration tank of the iWWTP had been shut down on 14 October 2022 and the maximum incubation period of 14 days was exceeded. This aeration tank remained shut down until the tank could be fully covered on 7 April 2023.

## Discussion

We describe here a multidisciplinar­­y outbreak investigation that led to the identification and elimination of a lesser-known source of *Legionella* infection. Microbiological results and statistical modelling suggested the iWWTP and mWWTP as potential sources. The outbreak came to a direct halt when measures were taken at the iWWTP, suggesting that at least one of the plants, but probably both, were the infection source in this outbreak.

This study shows the added value of whole genome sequencing to discriminate between outbreak isolates, especially for STs that are common in the environment. Indeed, it has been increasingly used in *Legionella* outbreak investigations in the past decade [27-29]. Based on sequence typing and cgMLST, one in three cases with available clinical isolates matched with an isolate from the mWWTP that tested positive for *L. pneumophila* sg1 ST42 but did not match an isolate from the iWWTP. Outbreaks with multiple ST types are relatively common in *Legionella* outbreaks, and often, only some of the ST types that are present environmental sources can be confirmed [27,28,30]. A possible explanation could be that the causative, possibly more virulent, strain is present in low concentrations, while other strains are abundantly present in the WWTP. Hence, the latter are more likely to be detected, as has been reported previously [31]. Furthermore, multiple STs may form a mixed culture, and picking from such cultures may lead to isolation of only one of those STs. Repeated sampling and typing may therefore be required to find the outbreak strain in the source. Although the patients with ST82 were epidemiologically linked the outbreak, they could not be microbiologically linked to a source. Interestingly, our patient isolates were also closely related by cgMLST to ST82 isolates from patients (n = 3) and the environment (n = 1) not connected to this outbreak. Indeed, previous studies reporting on the genomic population structure of *Legionella* isolates have made similar observations for some ST types, indicating that isolates may be genetically closely related but not epidemiologically linked [32-34]. Of interest, on 18 and 25 October 2022, one effluent and one aeration tank sample taken by third parties at the iWWTP tested positive for *L. pneumophila* sg1 and sg2, with concentrations of 1,090,000 cfu/L and 200,000 cfu/L, respectively, but with unknown ST types (laboratory reports from the iWWTP; personal communication: Henry van Herwijnen, October 2022). This confirmed the continued draining of *Legionella*-contaminated water to the mWWTP, despite the shutdown of the iWWTP aeration tank, and showed the need for the UV-disinfection of the effluent as a control measure.

The use of statistical models based on wind direction, taking into account the probability-weighted incubation time and wind velocity, has to our knowledge not been applied before in LD outbreaks. While the outcomes of these models alone may not be sufficient for shaping control measures, the collective evidence, including spatial source identification models and environmental investigations, has played a crucial role in pinpointing the most probable source. Here, model results indicated that mainly the mWWTP was in line with the upwind direction of the patients’ residential address, suggesting that the mWWTP potentially played a larger role than the iWWTP. This could possibly be due to the larger aeration volume, causing more aerosol formation. However, this does not exclude a role of aerolisation of *Legionella* at the iWWTP in this outbreak, as the outbreak strain might not have been detected in the samples taken at the iWWTP, e.g. because it was present in low concentrations [31]. The mall was also significantly associated with the upwind direction. However, this was considered an unlikely source of infection because only a limited number of patients were exposed to the supermarket misting system, which was the only identified possible source of infection in the mall. Moreover, *Legionella* was not detected in samples taken from the misting system.

The iWWTP identified in this outbreak had introduced an anaerobic treatment for biogas production 2 years before the outbreak, which was temporarily shut down and restarted in the year before the outbreak because of operational difficulties. The shutdown of the anaerobic treatment step reduced the efficiency of the treatment process, which led to higher concentrations of amino acids and nutrients in the warm wastewater, an identified risk for increased *Legionella* growth [35-37]. Similar changes in the treatment process were also observed in two previous WWTP outbreaks in the Netherlands, where both sites added an anaerobic treatment for biogas production about 1 year before the outbreak [10,11]. The combination of an anaerobic treatment, commonly requiring optimal operational temperatures for *Legionella* proliferation from 30–38 °C, followed by aerobic treatment, is a potential risk factor for rapid *Legionella* growth in a WWTP and dispersion to the environment [6,36]. Moreover, these systems are mostly used to process wastewater that is rich in proteins and amino acids, further promoting *Legionella* growth [35-37]. Indeed, the measured operating temperature of the iWWTP ranged from 30 °C during winter to 38 °C in summer, while the low operating temperature of the mWWTP (below 25 °C) was unlikely to promote rapid growth of *Legionella*. However, based on the outcome of the analyses, we assume that the mWWTP may have played an important role in the dispersion of *Legionella* due to its larger aeration volume, while the iWWTP was most likely the primary source of *Legionella* growth that contaminated the influx of the mWWTTP. This highlights the importance of taking into account the influent of potentially *Legionella-*contaminated water in the risk analysis of a WWTP, even for WWTPs that operate at temperatures that are too low for rapid *Legionella* growth. This is corroborated by previous studies that could not find a clear association between environmental factors, such as temperature, and the presence of *Legionella* in WWTPs [6]. 

Taking control measures to prevent *Legionella* in biological WWTPs is challenging. Unlike wet cooling towers, use of biocides is not possible in the biological treatment process, and draining a WWTP for cleaning and disinfection poses a risk as this may cause contamination of the surface water or the mWWTP to which the water is drained to. In this outbreak, full coverage of the aeration tank was difficult because of the large area and aeration volume of the tank, which may cause overpressure under the cover, requiring air extraction with air disinfection. Furthermore, a complete cover may increase the temperature in an aeration tank which may actually promote the growth of *Legionella*. Other measures that may be considered for some WWTPs are reducing aerosol formation by changes to the aeration system or using a floating cover, although the effectiveness of such control measures will need to be evaluated.

This study has several limitations. Firstly, typing information was only available for three cases, which hampered matching of human cases with typing information from environmental samples. Most LD patients are diagnosed with urine antigen testing or PCR, and a sputum sample for culture is often not available because patients do not have a productive cough. Secondly, both models used the residential addresses of the patients as model input, while cases could have been exposed to *Legionella* at another location in Houten. However, a previous study showed that spatial exposure mostly occurs at the residential address [23]. Thirdly, we used data on average daily wind direction, while it may have changed during the day. Lastly, information on smoking status was not available for six of 15 patients, which was probably not recorded because they had other underlying health conditions. This could also explain the low number of current smokers among the patients. This outbreak had an unusual male:female ratio with nine females and six males, while usually around 70% of LD patients are male [13]. However, no possible explanation could be found.

## Conclusion

This *Legionella* outbreak underlines the potential of municipal and industrial WWTPs to cause community cases and outbreaks of LD, especially those with favourable conditions for *Legionella* growth and dissemination, or even non-favourable conditions for growth but with the influx of contaminated water. This is particularly important because not all public health professionals may be aware of the LD risk of WWTPs, illustrated by fact that is not named as a source in the ECDC LD-GIS-tool. An inventory of these potential sources should be readily available for public health authorities to enable a rapid source outbreak investigation in case of a community cluster of Legionnaires’ disease. Furthermore, conducting risk analysis of WWTPs could aid in identifying those at increased risk for *Legionella* proliferation, thereby enabling of preventive measures.
